# Mental Simulations of Phonological Representations Are Causally Linked to Silent Reading of Direct Versus Indirect Speech

**DOI:** 10.5334/joc.141

**Published:** 2021-01-08

**Authors:** Bo Yao

**Affiliations:** 1 Division of Neuroscience and Experimental Psychology, Faculty of Biology, Medicine and Health, University of Manchester, UK

**Keywords:** Sentence processing, Semantics, Reading, Eye movements, Embodied cognition, Discourse processing

## Abstract

Embodied theories propose that language is understood via mental simulations of sensory states related to perception and action. Given that direct speech (e.g., *She says, “It’s a lovely day!”*) is perceived to be more vivid than indirect speech (e.g., *She says (that) it’s a lovely day*) in perception, recent research shows in silent reading that more vivid speech representations are mentally simulated for direct speech than for indirect speech. This ‘simulated’ speech is found to contain suprasegmental prosodic representations (e.g., speech prosody) but its phonological detail and its causal role in silent reading of direct speech remain unclear. Here in three experiments, I explored the phonological aspect and the causal role of speech simulations in silent reading of tongue twisters in direct speech, indirect speech and non-speech sentences. The results demonstrated greater visual tongue-twister effects (phonemic interference) during silent reading (Experiment 1) but not oral reading (Experiment 2) of direct speech as compared to indirect speech and non-speech. The tongue-twister effects in silent reading of direct speech were selectively disrupted by phonological interference (concurrent articulation) as compared to manual interference (finger tapping) (Experiment 3). The results replicated more vivid speech simulations in silent reading of direct speech, and additionally extended them to the phonological dimension. Crucially, they demonstrated a causal role of phonological simulations in silent reading of direct speech, at least in tongue-twister reading. The findings are discussed in relation to multidimensionality and task dependence of mental simulation and its mechanisms.

## 1. INTRODUCTION

As we immerse ourselves in a good book, our brains often create imaginative worlds with vivid and complex experiences. One such experience is fictional characters’ voices which can be so realistic and engaging that they bring the story to life (Alderson-Day, Bernini, & Fernyhough, 2017). Although many of us do share the intuition of hearing an “inner voice” or inner speech during silent reading, this mental phenomenon remains underspecified (Vilhauer, 2016).

Inner speech is an audible speech-like experience in the absence of an external speaker. Research has reported many perceptual similarities between *reading-induced* inner speech and overt speech. At the word level, phonetically longer words were slower to recognise during visual lexical decision (e.g., response times to *sw**a**n* or ***h**ate* are slower than *sw**a**p* or ***c**ape*), which resembled vowel- and consonant-length effects in overt speech (Abramson & Goldinger, 1997). Similarly, words with more stressed syllables were fixated on for longer in silent reading (e.g., ***fun**da**men**tal* takes longer to read than *sig**ni**ficant*), suggesting that low-level phonetic properties of speech were mentally represented during sentence reading (Ashby & Clifton, 2005). When reading limericks, northern or southern English speakers in England experienced rhyming effects that were specific to their own accents (e.g., *Bath* rhymes with *Kath* in a northern accent but rhymes with *Garth* in a southern accent), showing that inner speech retained higher-level auditory properties of one’s accent (Filik & Barber, 2011). At the discourse level, readers were found to mentally imagine a talker’s voice when they were reading a passage that was presumably ‘written’ by that talker (Alexander & Nygaard, 2008) or when reading familiar scripts that were previously heard (Kurby, Magliano, & Rapp, 2009), indicating the involvement of talker-specific speech representations during silent reading. Since these perceptual properties are either introduced by low-level phonological processing or introduced by prior exposure to specific voices, this kind of *‘induced’* inner speech does not necessarily depend on language comprehension.

More recent research, however, suggests that some inner speech can be viewed as *mental simulations* of speech and an integral part of language comprehension in silent reading (Yao & Scheepers, 2015). Embodied theories propose that language is understood via mental simulations of sensory states related to perception and action (Barsalou, 1999; Barsalou, 2008; Zwaan, 2004). Under this framework, written dialogues should be understood in mental simulations of more vivid vocal depictions when dialogues are reported directly (e.g., *She says, “It’s a lovely day!”*) than when they are reported indirectly (e.g., *She says (that) it’s a lovely day*) (Clark & Gerrig, 1990; Yao, 2011). To test this prediction, Yao and colleagues (2011) used eye tracking and fMRI to compare neural activity in silent reading of direct vs. indirect speech. While both kinds of reported speech activated the auditory cortex, direct speech was associated with greater neural activity in areas of the auditory cortex that selectively responded to human voice (Belin, Zatorre, Lafaille, Ahad, & Pike, 2000). The findings suggest that a more vivid inner speech is mentally activated during silent reading of direct rather than indirect speech. Importantly, this more vivid inner speech depends on language comprehension. For example, direct (but not indirect) speech quotations are read faster when they are preceded by descriptions indicating a fast speaking rate (e.g., *He said quickly vs. He said slowly*), suggesting that the speed of inner speech is influenced by semantic processing (Stites, Luke, & Christianson, 2013; Yao & Scheepers, 2011). Listening to monotonously spoken direct (vs. indirect) speech elicits greater neural activity in the voice-selective areas, suggesting that more vivid prosody (e.g., a joyful intonation) is mentally generated in a top-down fashion for monotonous, prosody-impoverished direct speech; this more vivid inner prosody is what one expects to hear based on comprehension of the given scenario (e.g., friends talking about an enjoyable experience at a theatre) (Yao, Belin, & Scheepers, 2012). Thus, unlike inner speech that is primarily induced by voice exposure or intentional imagery, this more vivid inner speech is more spontaneously *‘simulated’* (i.e. mentally re-enacted without conscious intentions) as part of language comprehension.

The research on simulated inner speech contributes to embodied theories of language in two respects. Theoretically, it highlights that mental simulations are not only driven by semantic processing, but are also influenced by language pragmatics (how language is used in context); semantically-matched sentences can be simulated differently when they are reported in styles that are associated with different perceptual experiences. Methodologically, this research advocates the use of eye tracking and/or fMRI to study mental simulations in normal reading conditions. Such paradigms can ensure that simulation effects are observed naturally during online language comprehension and are not driven or probed offline by secondary tasks seen in classic paradigms (e.g., the sentence-picture-verification task; cf. Zwaan, Stanfield, & Yaxley, 2002).

The current evidence of simulated inner speech, however, is by no means conclusive. Two challenges remain. First, the perceptual detail of simulated inner speech is not fully characterised. To understand *how* mental simulation works, we must first identify *what* perceptual features are being simulated. Although evidence suggests that simulated inner speech contains suprasegmental prosodic information (Stites et al., 2013; Yao & Scheepers, 2011; Yao et al., 2012), it remains unknown whether it also consists of segmental phonological features. In previous fMRI studies (Yao et al., 2011, 2012), comprehension of direct (vs. indirect) speech elicited greater neural activity in the right auditory cortex for prosodic processing, but not in the left auditory cortex typically associated with phonological processing (Hickok & Poeppel, 2007; Scott, Blank, Rosen, & Wise, 2000). The lack of direct speech effects on phonological activation may suggest that simulated inner speech is predominantly prosodic in nature. Compared to low-level phonological representations, prosody is more critical for making inferences about the protagonist’s mental states and intentions (Graesser, Singer, & Trabasso, 1994; Scott, 2019) and therefore may be more robustly simulated in text comprehension (cf. Zwaan & Pecher, 2012 for similar arguments). However, the absence of evidence is not evidence of absence. Given that phonological processing is common in silent reading (Rayner, 1998), the phonological differences between direct and indirect speech may be too subtle to cause significant changes in blood-oxygen-level-dependent (BOLD) signals in relevant brain areas. More sensitive measures such as eye movements may be more effective in detecting subtle differences in phonological processing and address whether more vivid phonological detail is also simulated in silent reading of direct speech. Second, the causal consequence of simulated inner speech on language comprehension is yet to be established. Evidence obtained using eye tracking and fMRI is *correlational*. It demonstrates that silent reading of direct speech is associated with simulated inner speech, but not necessarily depends on it. For example, the effects of direct speech on mental models has been questioned by Eerland, Engelen and Zwaan (2013). They showed that readers focused more on exact wording during silent reading of direct (vs. indirect) speech but did not construct more accessible situation models (Morrow, Greenspan, & Bower, 1987) for referential or communicative situations.

The present paper addresses the above challenges by (1) probing the phonological aspect of simulated inner speech and (2) testing the causal role of simulated inner speech in silent reading of direct (vs. indirect) speech. Given that phonologically detailed imagery is observed in silent reading (Filik & Barber, 2011; Kurby et al., 2009), it is reasonable to hypothesise that simulated inner speech, which is phenomenologically similar to auditory imagery (Alexander & Nygaard, 2008; Yao & Scheepers, 2011), also contain detailed phonological representations. Importantly, simulations of such phonological representations should be *causally* linked to language processing of direct speech in particular, if an embodied account of simulated inner speech were to be accepted (Ostarek & Huettig, 2019).

One way to quantify phonological simulations in silent reading of direct vs. indirect speech is by measuring the ‘visual tongue-twister’ or ‘phonemic interference’ effect (McCutchen, Bell, France, & Perfetti, 1991; McCutchen & Perfetti, 1982). In McCutchen and Perfetti (1982)’s seminal study, sensibility judgements on tongue twisters (e.g., ***T**wenty **t**oys were in the **t**runk*) took longer than on matched non-tongue twisters (e.g., ***S**everal **g**ames were in the **ch**est*). Longer reading times on tongue twisters reflected increased interference between repeated word-initial phonemes that are activated during silent reading. If more detailed phonological representations are simulated in silent reading of direct rather than indirect speech, greater visual tongue-twister effects (longer reading times) should be observed when tongue twisters are embedded in direct rather than indirect speech. If such phonological simulations are causally linked to silent reading of direct speech, the latter should be selectively disrupted by phonological interference, resulting in a larger reduction in visual tongue-twister effects (shorter reading times).

The above predictions were tested in three experiments. English tongue twisters were embedded in direct speech, indirect speech or non-speech sentences. Participants read these sentences either silently (Experiment 1 & 3) or orally (Experiment 2). Using eye tracking, Experiment 1 examined visual tongue-twister effects (measured by first-pass reading times) between silent reading of direct speech, indirect speech and non-speech sentences. Experiment 2 tested whether the direct speech effects on tongue twister reading in Experiment 1 were phonological in nature, by maxing out and equalising phonological details across conditions in overt articulation. Finally, Experiment 3 tested the causal role of phonological simulations in silent reading of direct vs. indirect speech by disrupting tongue-twister reading with phonological vs. manual interference. In all three experiments, participants gave written informed consent and the experimental procedure was approved by the University Research Ethics Committee at the University of Manchester (Ref 16248).

## 2. EXPERIMENT 1

### 2.1. PARTICIPANTS

Fifty-four native speakers of English with no reported reading impairments took part in the study in exchange for £3. A typical session lasted about 30 min. In total, three participants were excluded from analysis due to excessive blinks (1 participant) or less than 70% comprehension accuracy (2 participants), leaving 51 participants (33 female, *Mean*_age_ = 24.5, *SD*_age_ = 8.3) for analysis.

### 2.2. MATERIALS AND DESIGN

Twenty-four triplets of fictional stories were written in the present tense as reading materials (see ***Appendix A***). Each triplet consisted of two common background sentences (sentence 1 & 2) and one common ending (sentence 4). Sentence 3 of the stories reported an identical tongue twister in either direct speech (DS), indirect speech (IS), or a non-speech (NS) sentence (see ***Table 1***). This resulted in a one-factor (Reporting Style) within-subject design with three levels.

**Table 1:** Example stimulus item with three conditions in Experiment 1.


*Giovanni De Luca owns an independent Italian coffee shop that proves very popular in Didsbury. Entrepreneur Alexander J. Jones wants to invest in Giovanni’s coffee and asks him his coffee-making secret*.	

**DS**	*Giovanni laughs and explains, “You can only make a proper cup of coffee from a proper copper coffee pot.”*

**IS**	*Giovanni laughs and explains that one can only make a proper cup of coffee from a proper copper coffee pot.*

**NS**	*Giovanni laughs and shows him that one can only make a proper cup of coffee from a proper copper coffee pot*.

*The entrepreneur realises that he needs more cash to invest in these copper coffee pots*.	


DS = direct speech; IS = indirect speech; NS = non-speech.

The critical stories were mixed with 12 filler stories containing no tongue twisters (see ***Appendix A***). Experimental and filler items spanned 4 lines on screen, with the critical tongue twisters consistently positioned at the centre of line 3 across conditions. A total of 12 items were accompanied with yes/no comprehension questions to encourage reading for comprehension.

The reading materials were counterbalanced using a Latin square before being allocated to three stimulus lists (8 critical items per condition per list). Each list was assigned to 18 participants.

### 2.3. PROCEDURE

The experiment was conducted using a SR-Research EyeLink 1000 desk-mounted eye-tracking running at 1000 Hz sampling rate. Stimulus presentation was implemented in EyeTrack 0.7.10 m (University of Massachusetts Eyetracking Lab). Participants were seated about 70 cm from an LCD display running at 60 Hz refresh rate in 1650 × 1050 pixel resolution. Materials were presented in a 20pt Calibri font printed in black over a light grey background. Line spacing was set to 30 pts such that the fixation locations could be unambiguously mapped onto a corresponding line of text. Viewing was binocular, but only the dominant eye was tracked. A chin rest and a forehead rest were used to stabilise participants’ head position and keep viewing distance constant. Button responses were collected using a hand-held Microsoft USB game controller.

The experiment began with a 9-point calibration and validation procedure. Participants always saw 2 filler trials at the start of the experiment. The rest of the trials were presented in a random order. Each trial began with a gaze trigger which was located at the upper left side of the monitor. A fixation on this triggered the presentation of the passage. Participants read the passage silently and at their own pace. They pressed the X button on the controller to terminate the text presentation, which either triggered the next trial or a yes/no comprehension question regarding the story they had just read. Participants answered the questions using the left (‘yes’) or the right (‘no’) response button. Answering the question triggered the next trial.

### 2.4. RESULTS AND DISCUSSION

Fixation coordinates were mapped onto character and line positions using EyeDoctor 0.6.5. Fixations below 80 ms were pooled with temporally adjacent fixations if the latter were within half a degree of visual angle. Fixations above 1200 ms were truncated. Fixations within the critical tongue-twister regions (e.g., underscored in ***Table 1*** and in bold text in ***Appendix A***) were summarised in first-pass reading time (fpRT; the total fixation times from entering the critical region for the first time until leaving the region). Trials with missing values due to blink or tracking loss were removed (0.74% data loss). The mean fpRT and SDs across the three conditions are presented in ***Table 2***.

**Table 2:** Mean first-pass reading times and SDs (in ms) across experimental conditions in Experiment 1.


REPORTING STYLE	*N*	MEAN FPRT	*SD*

**Direct Speech**	407	2079	1290

**Indirect Speech**	404	1859	1241

**Non-Speech**	404	1867	1230


N = no. of observations; fpRT = first-pass reading time; SD = standard deviation.

**Figure 1:**
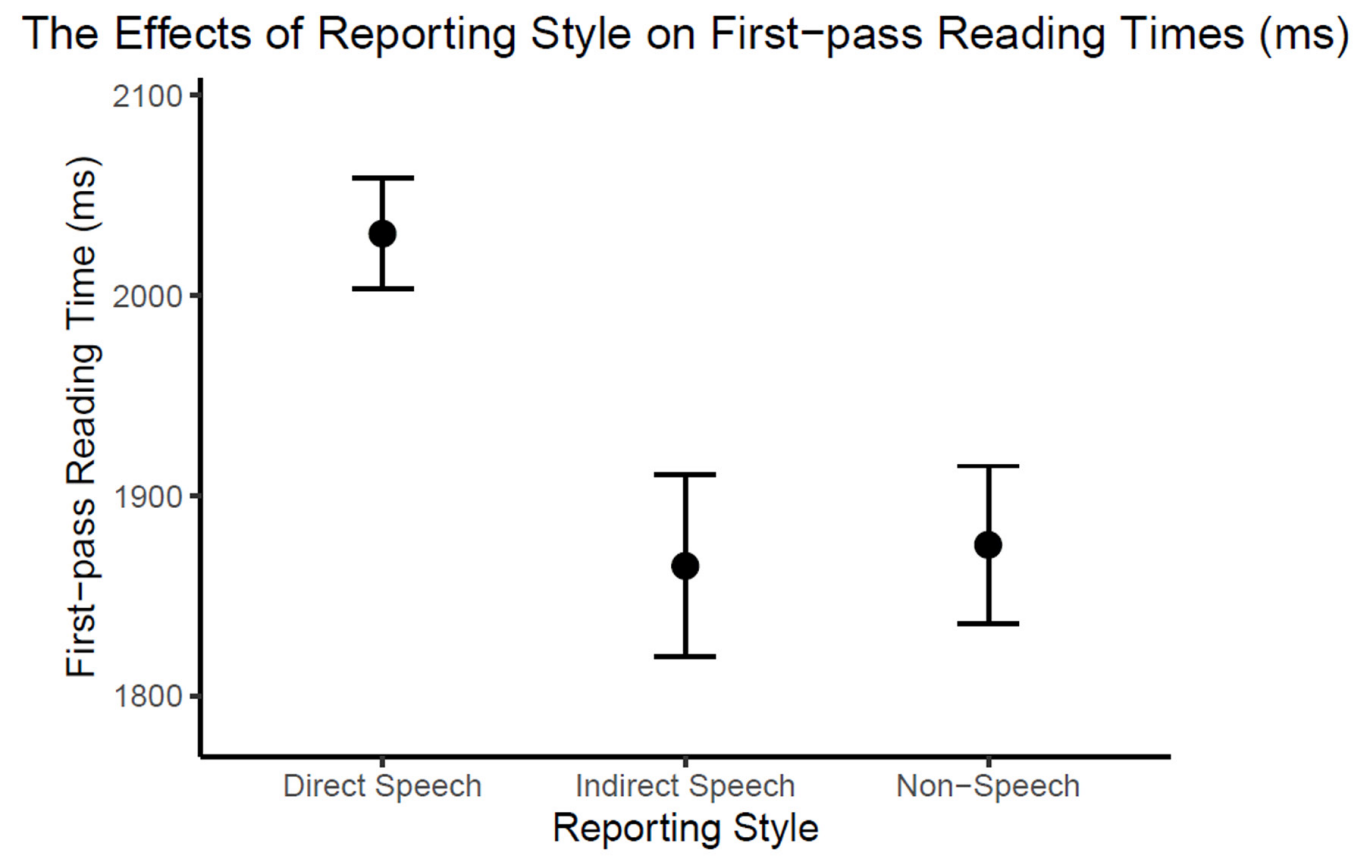
Model-estimated effects of Reporting Style on first-pass reading times in Experiment 1. The error bars represent 95% confidence intervals.

As fpRTs were positively skewed (skewness = 0.77), they were fitted in a generalised linear mixed model with a gamma distribution using the *glmer* function in *lme4* package in R. The three conditions were deviation-coded (mean centred and rescaled) into two contrast variables, with the *direct speech* (DS) condition as the baseline: The first variable encoded the *indirect speech* (IS)*-direct speech* (DS) contrast; the second the *non-speech* (NS)-*direct speech* (DS) contrast. Maximal random effect structure was employed with *subject* and *item* as crossed random factors, including all relevant random intercepts and slopes. The model estimates are reported in ***Table 3***, with between-condition contrasts illustrated in ***Figure 1***.

**Table 3:** Generalised linear mixed-effect model estimates of first-pass reading times for Experiment 1.


FIXED EFFECTS	*B*	*S.E.*	*T*	*P*

Intercept	2031	14.1	143.9	<.001

IS – DS	–166	20.2	–8.2	<.001

NS – DS	–156	14.6	–10.7	<.001


DS = Direct Speech; IS = Indirect Speech; NS = Non-Speech. P-values were calculated using Satterthwaite approximations (lmerTest package).

The results revealed that tongue-twister reading times were significantly longer in direct speech than in indirect speech (+166 ms) or non-speech (+156 ms). The reading time differences could not be attributed to different sentence lengths or grammatical tense because the critical tongue twisters were identical across conditions and were all in the present tense. The findings support the prediction of longer silent reading times on tongue twisters that are reported in direct speech as opposed to indirect speech and non-speech sentences.

However, longer reading times in direct speech may not necessarily be driven by increased phonemic interference. They could also be explained by higher, non-phonological cognitive demands on perspective taking (Köder, Maier, & Hendriks, 2015) and/or verbatim memory encoding (Eerland et al., 2013) in direct speech. Although the “phonemic interference” and “cognitive demand” accounts both seem consistent with our results, they would make different predictions in oral reading. If longer reading times in silent reading of direct speech were driven by increased phonemic interference, it would mean that more detailed phonological representations were activated in direct speech than in indirect speech and non-speech. The relative differences in phonological details would be evened out in oral reading because phonological representations would be fully activated for articulation and are maxed out at the ceiling level across all three conditions. Although the phonemic interference effects were relatively stronger in silent reading of direct speech, they would be *equally* pronounced in oral reading, regardless of reporting style. In contrast, if longer reading times in silent reading of direct speech were driven by higher cognitive demands in non-phonological domains, these relative differences should remain unaffected by increased phonological processing in oral reading. As such, we should still observe longer reading times in oral reading of direct speech, as compared to indirect speech and non-speech. Thus, to test whether the direct speech effects in Experiment 1 were phonological in nature, Experiment 2 maxed out phonological activations in oral reading, thereby equalising the levels of phonological details between conditions. If oral reading times were no longer different between reporting styles, it would demonstrate that the direct speech effects on tongue twister reading were indeed sensitive to phonological manipulation. If oral reading times remained significantly slower in direct speech, it would suggest that the direct speech effects in Experiment 1 would be more plausibly explained by non-phonological cognitive differences between direct speech and the other conditions.

## 3. EXPERIMENT 2

### 3.1. PARTICIPANTS

Twenty-four native speakers of English (11 female, *Mean*_age_ = 29.5, *SD*_age_ = 11.2) with no reported reading impairments took part in this experiment in exchange for 1 course credit or £2. The experiment took less than 15 minutes. A smaller sample was used in accordance with previous research (Yao & Scheepers, 2011) which showed that the direct speech effects on reading times are more robust in oral reading.

### 3.2. MATERIALS AND DESIGN

The design was the same as Experiment 1 but only the 24 critical items were included in Experiment 2. Fillers were not included because they could no longer conceal the experiment’s focus on tongue twisters which became instantly apparent during oral reading. The critical stimuli were presented in the same manner as in Experiment 1. The reading materials were counterbalanced using a Latin square before being allocated to three stimulus lists (8 critical items per condition per list). Each list was assigned to 8 participants.

### 3.3. PROCEDURE

Stimulus presentation and audio recording were implemented in OpenSesame (Mathôt, Schreij, & Theeuwes, 2012) on a Dell Optiplex lab computer. The presentation used a 1024 × 768 pixel resolution. Stimuli were presented in black text (18 px, Arial font) over a light grey background.

Each trial started with the string “NEW STORY” at the centre of the screen for 1000 ms, followed by the story. Participants were instructed to read the presented story out loud, and as fluently as possible. Their oral reading was recorded with a microphone in front of them. After reading each story, they would press the spacebar on a keyboard to proceed to the next.

### 3.4. RESULTS AND DISCUSSION

Speech recordings were listened to and coded by the experimenter. First-pass oral reading times on the critical tongue twisters were measured using GoldWave (*www.goldwave.com*). Here first-pass reading was defined as the first attempt in tongue-twister reading. One trial (0.2%) was excluded from the analysis as it was accidentally skipped by the participant. Trials with speech errors (hesitation, word repetition/omission, self-correction and incompletion) were included in the analysis because the errors were reflected in variation in reading times. The mean fpRT and SDs across the three conditions are presented in ***Table 4***.

**Table 4:** Mean first-pass oral reading times and SDs (in ms) across experimental conditions in Experiment 2.


REPORTING STYLE	*N*	MEAN FPRT	*SD*

**Direct Speech**	191	3074	1159

**Indirect Speech**	192	3214	1331

**Non-Speech**	192	3095	972


N = no. of observations; fpRT = first-pass reading time; SD = standard deviation.

**Figure 2:**
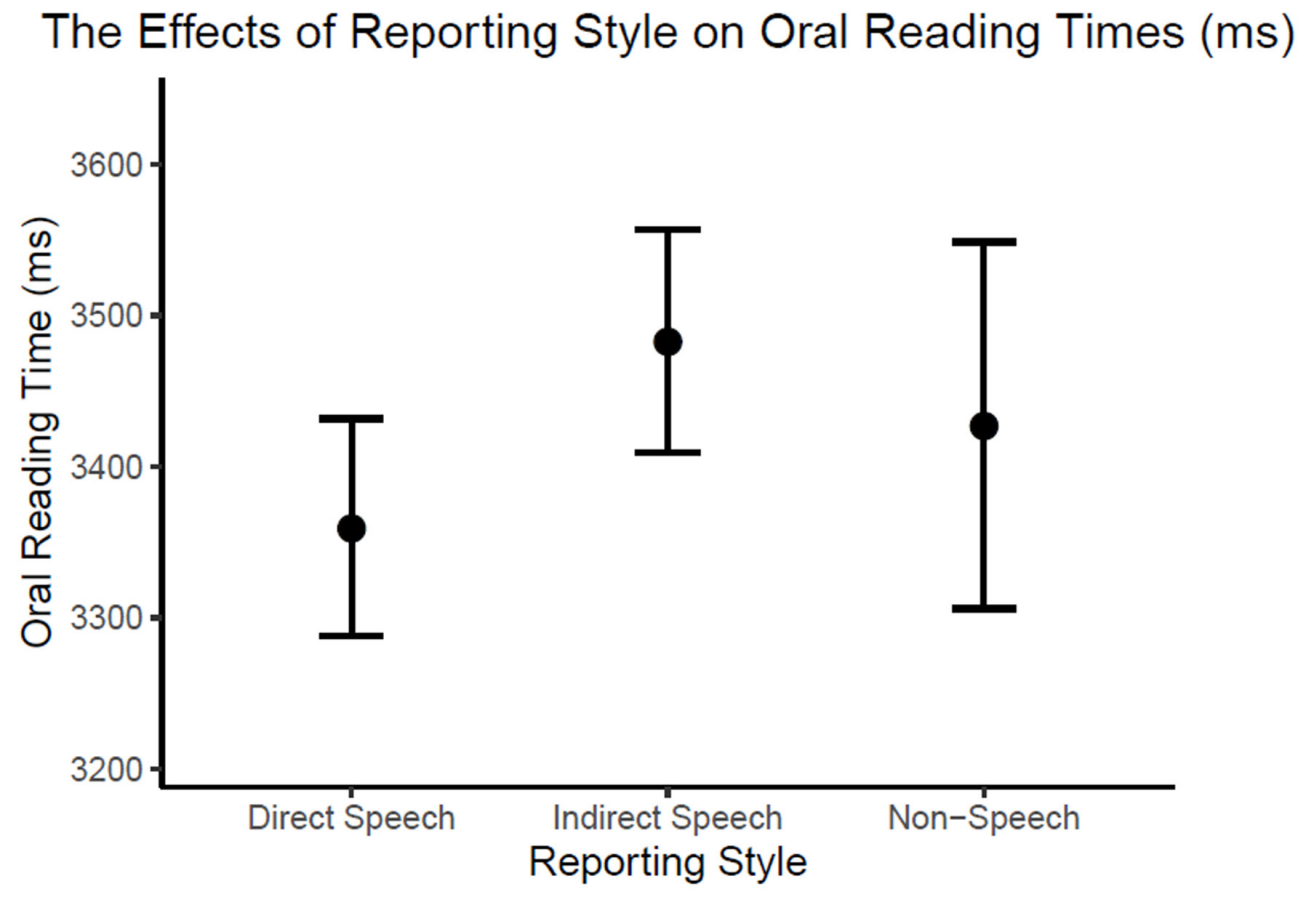
Model-estimated effects of Reporting Style on first-pass oral reading times in Experiment 2. The error bars represent 95% confidence intervals.

First-pass oral reading times were fitted in a generalised linear mixed model with a gamma distribution. The fixed and random effect structures were identical to Experiment 1. The model estimates are reported in ***Table 5***, with between-condition contrasts illustrated in ***Figure 2***.

**Table 5:** Generalised linear mixed-effect model estimates of first-pass oral reading times for Experiment 2.


FIXED EFFECTS	*B*	*S.E.*	*T*	*P*

Intercept	3424	37.8	90.6	<.001

IS – DS	116	26.7	4.3	<.001

NS – DS	64	47.7	1.3	.181


DS = Direct Speech; IS = Indirect Speech; NS = Non-Speech. P-values were calculated using Satterthwaite approximations (lmerTest package).

In line with the reading literature (Rayner, 1998), oral reading of tongue twisters (intercept = 3424 ms) was overall slower than silent reading of the same sentences (intercept = 2031 ms). Unlike the silent reading time results in Experiment 1, oral reading times were in fact shorter for direct speech than for indirect speech (–116 ms; *p* < .001) and for non-speech (–64 ms; *p* = .181). This speaks against the “cognitive demand” account which predicted longer reading times for direct speech due to its higher demands on perspective taking and/or memory load. That said, the shorter reading times for direct than indirect speech remained somewhat unexpected because the “phonemic interference” account predicted no reading time differences between them. One possible explanation for this finding may be provided by differential prosodic grouping in oral reading of direct vs. indirect speech. The use of punctuation and interjections in direct speech means that readers may be more likely to pause before direct-speech quotations than before indirect-speech quotations (e.g., *Giovanni laughs and explains*, [Pause] *“You can only make …”* vs. *Giovanni laughs and explains that one can only make …*) (Yao & Scheepers, 2018). As a result, direct speech may often be split into two shorter prosodic units which are easier to read orally than a long, continuous indirect speech sentence. Exploratory analysis confirmed higher rate of pauses^1^ before direct-speech quotations (68%) than indirect-speech quotations (28%) and non-speech sentences (19%). However, tongue twister reading times preceded by pauses (3364 ms) were on average slower than those without pauses (2983 ms), indicating that pauses may not necessarily make subsequent tongue twisters ‘easier’ to read. Interestingly, oral reading time differences between direct speech vs. indirect speech and non-speech were more pronounced in trials with pauses (3163 ms vs. 3802 ms vs. 3445 ms) than in trials without pauses (2885 ms vs. 2989 ms vs. 3015 ms). It suggests that some of the pauses may reflect anticipated difficulty in reading the upcoming tongue twisters, which may be higher in indirect speech and non-speech sentences due to their more complex subordinate clause structures. Regardless of what the causes might be for the shorter oral reading times in direct than indirect speech, the current results are more compatible with the “phonemic interference” account than the “cognitive demand” account. To provide further support for the “phonemic interference” account and test the causal role of increased phonological activation in silent reading of direct speech, Experiment 3 examined how silent reading of tongue twisters in direct vs. indirect speech may be differentially disrupted by concurrent phonological vs. manual interference.

## 4. EXPERIMENT 3

### 4.1. PARTICIPANTS

Fifty-two native speakers of English (38 female, *Mean*_age_ = 21.4, *SD*_age_ = 4.3) with no reported reading impairments took part in the study in exchange for £3. A typical session lasted about 30 minutes.

### 4.2. MATERIALS AND DESIGN

The materials (24 critical items and 12 fillers) and comprehension questions were identical to those in Experiment 1. The experimental design was changed in two ways. First, the ‘Reporting Style’ factor was simplified by removing the Non-Speech condition. Second, an ‘Interference’ factor was introduced in which half of the stimuli were assigned to the phonological interference condition and the other half to the manual interference condition. This resulted in a 2 (Reporting Style: Direct Speech vs. Indirect Speech) × 2 (Interference: Phonological vs. Manual) within-subject design. The reading materials were counterbalanced using a Latin square before being allocated to four stimulus lists (6 critical items per condition per list). Each list was assigned to 13 participants.

### 4.3. PROCEDURE

The experimental procedure was mostly identical to that in Experiment 1. The only difference was the introduction of concurrent interference tasks. Each testing session was split into two blocks. In the phonological interference block, participants silently read stories while verbally counting *‘one, two, three, four …’* in a recursive manner at ~160 words per minute. The chin rest was not used in this block to allow jaw movements. In the manual interference block, they silently read stories while tapping the desktop with their left hand in a recurring sequence of index finger, middle finger, ring finger and little finger, also at ~160 taps per minute. Prior to each block, participants practised the corresponding interference task until they felt comfortable with it. During each block, participants were allowed to engage the interference task only during reading, and to rest between trials. If they forgot to maintain the interference task or did not follow the task procedure properly (e.g., verbal counting gradually turning into mumbling or slowed down significantly; finger tapping did not follow the correct sequence, etc.), they were reminded by the experimenter to re-engage the task before the next trial started.

### 4.4. RESULTS AND DISCUSSION

Fixation data were pre-processed in the same way as in Experiment 1 and were summarised in first-pass reading time (fpRT). Trials with missing values due to blink or tracking loss were removed (0.89% data loss). The mean fpRT and SDs across the three conditions are presented in ***Table 6***.

**Table 6:** Mean first-pass reading times and SDs (in ms) across experimental conditions in Experiment 3.


INTERFERENCE	REPORTING STYLE	*N*	MEAN FPRT	*SD*

Phonological	Direct Speech	309	1474	833

Phonological	Indirect Speech	310	1418	965

Manual	Direct Speech	306	1896	1162

Manual	Indirect Speech	302	1715	1152


N = no. of observations; fpRT = first-pass reading time; SD = standard deviation.

**Figure 3:**
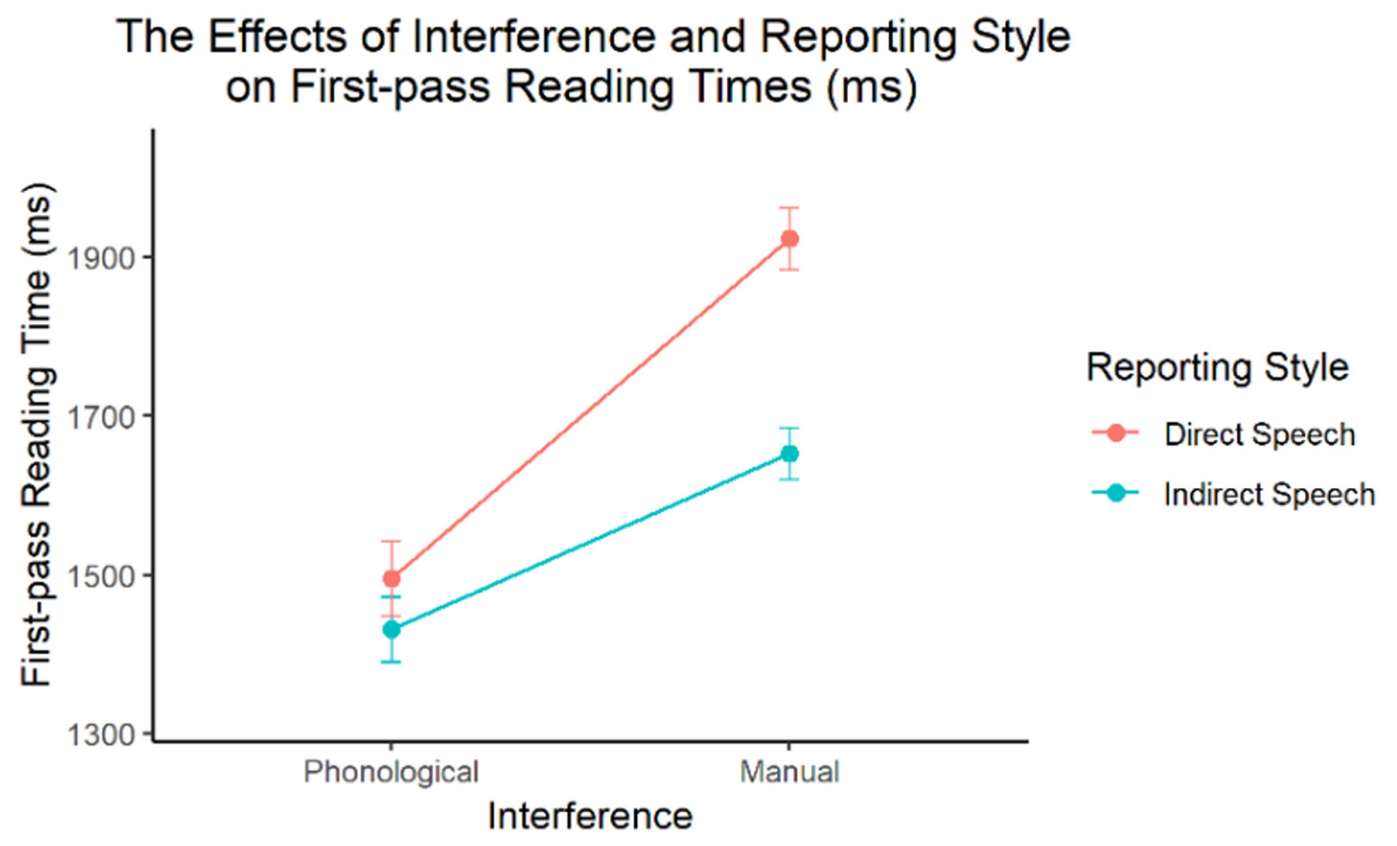
Model-estimated effects of Interference and Reporting Style on first-pass reading times in Experiment 3. The error bars represent 95% confidence intervals.

First-pass reading times were fitted in a generalised linear mixed model with a gamma distribution. The two fixed factors were deviation-coded into two contrast variables. The first variable encoded the *direct speech* (DS)-*indirect speech* (IS) contrast; the second variable encoded the *phonological interference* (PI)-*manual interference* (MI) contrast. Fixed effects structure included all main effects and the interaction between the two contrast variables. Maximal random effect structure was employed with *subject* and *item* as crossed random factors, including all relevant random intercepts and slopes. The model estimates are reported in ***Table 7***, with between-condition contrasts illustrated in ***Figure 3***.

**Table 7:** Generalised linear mixed-effect model estimates of first-pass reading times for Experiment 3.


FIXED EFFECTS	*B*	*S.E.*	*T*	*P*

**Intercept**	1624	15.1	107.3	<.001

**PI-MI**	–325	21.4	–15.2	<.001

**DS-IS**	166	16.4	10.1	<.001

**PI-MI x DS-IS**	–207	12.3	–16.8	<.001


PI = Phonological Interference; MI = Manual Interference; DS = Direct Speech; IS = Indirect Speech. P-values were calculated using Satterthwaite approximations (lmerTest package).

The results showed that silent reading times with interference (Experiment 3; intercept = 1624 ms) were shorter than those without (Experiment 1; intercept = 2031 ms). Reading times during phonological interference were significantly shorter than those during manual interference (–325 ms). These results do not support the “cognitive demand” account because interference techniques should increase overall cognitive demands, resulting in longer reading times. The shorter reading times are more compatible with the “phonemic interference” account and are more likely to reflect a reduction in visual tongue-twister effects. Critically, the reduction in visual tongue-twister effects was larger during articulation suppression as compared to finger tapping. Given that visual tongue-twister effects result from phonemic interference (McCutchen et al., 1991), this main effect indicates that articulation suppression is effective in disrupting phonological processing.

Consistent with Experiment 1, the main effect of Reporting Style was also significant, reflecting that tongue twisters in direct speech took longer to read than those in indirect speech (+166 ms). The central question here concerns how this Reporting Style effect was modulated by different interference techniques. There was indeed a significant interaction between the two factors: reading time differences between direct and indirect speech were significantly smaller during phonological interference (ΔfpRT = 64 ms, 95%CI = [31 ms 97 ms]) than during manual interference (ΔfpRT = 271 ms, 95%CI = [235 ms 306 ms]).

The findings support the hypothesis that more detailed phonological representations are activated in silent reading of direct than indirect speech. Crucially, more detailed phonological activation is causally linked to silent reading of direct speech because the latter can be selectively disrupted by phonological interference as opposed to manual, non-phonological interference.

## 5. GENERAL DISCUSSION

The present study demonstrated more pronounced visual tongue-twister (phonemic interference) effects during silent reading of direct speech as compared to indirect speech and non-speech sentences (Experiment 1). The observed reading time differences may be driven by more vivid phonological activation in direct speech, which were evened out between conditions in overt articulation where phonological representations were fully activated regardless of reporting styles (Experiment 2). Crucially, this increased phonological activation may be causally linked to silent reading of direct speech in particular, where tongue twister reading was selectively disrupted by phonological interference (concurrent articulation) rather than manual interference (finger tapping). The findings cannot be explained by differential phonological profiles between direct speech and other reporting styles because the critical tongue twisters were identical between conditions.

The current results are more plausibly explained within the embodied framework (Barsalou, 1999; Barsalou, 2008; Zwaan, 2004), in which language comprehension depends on mental simulations (i.e. re-enactments) of sensory states related to perception and action. The increased phonological activation in direct speech can be interpreted as mental simulations of *more detailed* phonological representations in addition to *‘default’* phonological processing in silent reading. This is consistent with the observations that direct speech is typically perceived with more vivid vocal depictions in oral communication (Clark & Gerrig, 1990; Yao, 2011) and the empirical evidence that silent reading of direct speech is associated with more vivid speech representations (Yao & Scheepers, 2011; Yao et al., 2011; Yao & Scheepers, 2015).

The present study advances the embodiment literature in two respects. First, it highlights the multidimensionality and potential task dependence of mental simulation in the auditory domain. Just as dimensions of orientation (Stanfield & Zwaan, 2001), shape (Zwaan et al., 2002) and colour (Connell, 2007) may be mentally stimulated in language comprehension of visual objects, it is perhaps not surprising that both phonological and prosodic representations are simulated for understanding written speech. What is surprising, however, is why phonological simulations were not observed in previous studies on simulated inner speech. Using fMRI, for instance, silent reading of direct (vs. indirect) speech was found to increase neural activity only in the right auditory cortex for prosodic processing (Yao et al., 2011, 2012), but not in the left auditory cortex typically associated with phonological processing (Hickok & Poeppel, 2007; Scott et al., 2000). On the one hand, the lack of evidence for phonological simulations may be attributed to the insensitivity of BOLD measure and nuanced fMRI data analyses, in that the phonological difference between direct and indirect speech may be too subtle to be captured in BOLD signal changes in the regions of interest and did not survive the statistical tests. On the other hand, a theoretical explanation is that mental simulation is *flexible* and may only occur when it is relevant for the task at hand (Ostarek & Huettig, 2017). While prosodic simulations may be more relevant for contextual, temporal and syntactic processing of written speech (Yao & Scheepers, 2011, 2018; Yao et al., 2012), phonological simulations may only become observable when the task places a high demand on phonological processing (e.g., tongue-twister reading). In this sense, the present study has not only addressed what auditory dimensions could be mentally simulated, but has also emphasised the need for specifying the task conditions and mechanisms under which sensory dimensions are variably simulated, integrated and observed in a given paradigm.

The second contribution of this study is testing the causality of phonological simulations in silent reading of direct speech. As highlighted by Ostarek and Huettig (2019), a key challenge facing embodiment research today regards whether observed embodied effects are epiphenomenal or causally linked to language processing. In the visual domain, it has been shown that concurrent visual noise can interfere with conceptual processing of visual information (Edmiston & Lupyan, 2017; Ostarek & Huettig, 2017). In a similar vein, the present study demonstrated that phonological (vs. manual) interference can selectively disrupt silent reading of tongue twisters in direct rather than indirect speech, suggesting a causal link between phonological simulations and silent reading of direct speech. Some caution is warranted here as it remains inconclusive whether phonological simulations affect language comprehension, or whether they affect phonological processing of reading independent of comprehension. For instance, phonological simulations may draw upon forward models of speech production (Oppenheim, 2013; Pickering & Garrod, 2013) which can be selectively disrupted by articulation suppression but do not necessarily contribute to comprehension *per se*. Nevertheless, the finding complements the existing literature which has only provided correlational evidence of speech simulations in silent reading. It also provides evidence that behavioural interference paradigms such as articulation suppression can be effective in probing the causality of mental simulation in language processing and should be used more widely to test embodied accounts of language and cognition.

Of course, there are open questions that remain to be addressed. Some are related to whether and how speech simulations may be governed by multiple mechanisms. Given that phonological and prosodic processing of overt speech are supported by distinct neural circuits (Scott et al., 2000; Wildgruber, Ackermann, Kreifelts, & Ethofer, 2006; Zatorre, Belin, & Penhune, 2002), simulations of these representations may also be implemented differently. For instance, a recent study (Tian, Zarate, & Poeppel, 2016) proposed two mechanisms for speech imagery – one relying on *articulatory simulation* and the other on *memory retrieval*; speech representations can either be estimated as perceptual consequences of articulatory simulation (motor preparation), or be reconstructed by stored perceptual memories in auditory cortices. In comparison, speech simulations could be governed by similar mechanisms: it may engage the articulatory system to simulate auditory features that can be produced by oneself, and may draw on the memory system to retrieve richer auditory details that characterise another talker’s identity and emotion for example. If mental simulation is supported jointly by multiple mechanisms, how are these mechanisms weighted by task demand and how do they interact to form a coherent mental representation? Are they contributing to simulation in a parallel and distributed network, or are they organised hierarchically and integrated in a hub-and-spoke model? Answering these questions could substantially deepen our understanding of an embodied simulation system, enabling us to make more precise predictions on embodied effects in language and cognition.

In conclusion, the present study investigated mental simulations of phonological representations in silent reading of direct vs. indirect speech. The results showed increased visual tongue-twister effects (longer tongue-twister reading times) in silent reading of direct rather than indirect speech, which were equalised in oral reading and were selectively reduced by concurrent phonological interference. The findings suggest that silent reading of direct speech in particular may causally depend on mental simulations of more detailed phonological representations. Together with previous findings of prosodic simulations in silent reading of direct speech, the present study highlights that mental simulation may be multidimensional, and suggests that sensory dimensions may be differentially simulated by multiple mechanisms, depending on task demands.

## DATA ACCESSIBILITY STATEMENT

All data and R script in the research will be archived at *https://reshare.ukdataservice.ac.uk/*, *10.5255/UKDA-SN-854460*.

## ADDITIONAL FILE

The additional file for this article can be found as follows:

10.5334/joc.141.s1 Appendix A. Reading Materials (24 critical items + 12 fillers).

## Footnotes

Pauses here are defined as silent periods that are equal to or longer than 300 ms.

## References

[1] Abramson, M., & Goldinger, S. D. (1997). What the reader’s eye tells the mind’s ear: Silent reading activates inner speech. Perception & Psychophysics, 59(7), 1059–1068. DOI: 10.3758/BF03205520 9360478

[2] Alderson-Day, B., Bernini, M., & Fernyhough, C. (2017). Uncharted features and dynamics of reading: Voices, characters, and crossing of experiences. Consciousness and Cognition, 49, 98–109. DOI: 10.1016/j.concog.2017.01.003 28161599 PMC5361686

[3] Alexander, J. D., & Nygaard, L. C. (2008). Reading voices and hearing text: talker-specific auditory imagery in reading. Journal of Experimental Psychology. Human Perception and Performance, 34(2), 446–459. DOI: 10.1037/0096-1523.34.2.446 18377181

[4] Ashby, J., & Clifton, C. (2005). The prosodic property of lexical stress affects eye movements during silent reading. Cognition, 96(3), B89–100. DOI: 10.1016/j.cognition.2004.12.006 15913592 PMC1479854

[5] Barsalou, L. W. (1999). Perceptual symbol systems. Behavioral and Brain Sciences, 22(4), 577–660. DOI: 10.1017/S0140525X99002149 11301525

[6] Barsalou, L. W. (2008). Grounded cognition. Annual review of psychology, 59, 617–645. DOI: 10.1146/annurev.psych.59.103006.093639 17705682

[7] Belin, P., Zatorre, R. J., Lafaille, P., Ahad, P., & Pike, B. (2000). Voice-selective areas in human auditory cortex. Nature, 403(6767), 309–312. DOI: 10.1038/35002078 10659849

[8] Clark, H. H., & Gerrig, R. J. (1990). Quotations as Demonstrations. Language, 66(4), 764–805. DOI: 10.2307/414729

[9] Connell, L. (2007). Representing object colour in language comprehension. Cognition, 102(3), 476–485. DOI: 10.1016/j.cognition.2006.02.009 16616075

[10] Edmiston, P., & Lupyan, G. (2017). Visual interference disrupts visual knowledge. Journal of Memory and Language, 92, 281–292. DOI: 10.1016/j.jml.2016.07.002

[11] Eerland, A., Engelen, J. A. A., & Zwaan, R. A. (2013). The influence of direct and indirect speech on mental representations. Plos One, 8(6), e65480 DOI: 10.1371/journal.pone.0065480 23776488 PMC3680483

[12] Filik, R., & Barber, E. (2011). Inner speech during silent reading reflects the reader’s regional accent. Plos One, 6(10), e25782 DOI: 10.1371/journal.pone.0025782 22039423 PMC3198452

[13] Graesser, A. C., Singer, M., & Trabasso, T. (1994). Constructing inferences during narrative text comprehension. Psychological Review, 101(3), 371–395. DOI: 10.1037/0033-295X.101.3.371 7938337

[14] Hickok, G., & Poeppel, D. (2007). The cortical organization of speech processing. Nature Reviews. Neuroscience, 8(5), 393–402. DOI: 10.1038/nrn2113 17431404

[15] Köder, F., Maier, E., & Hendriks, P. (2015). Perspective shift increases processing effort of pronouns: a comparison between direct and indirect speech. Language, cognition and neuroscience, 30(8), 940–946. DOI: 10.1080/23273798.2015.1047460

[16] Kurby, C. A., Magliano, J. P., & Rapp, D. N. (2009). Those voices in your head: activation of auditory images during reading. Cognition, 112(3), 457–461. DOI: 10.1016/j.cognition.2009.05.007 19540472 PMC2739653

[17] McCutchen, D., Bell, L. C., France, I. M., & Perfetti, C. A. (1991). Phoneme-specific interference in reading: The tongue-twister effect revisited. Reading Research Quarterly, 26(1), 87–103. DOI: 10.2307/747733

[18] McCutchen, D., & Perfetti, C. A. (1982). The visual tongue-twister effect: Phonological activation in silent reading. Journal of Verbal Learning and Verbal Behavior, 21(6), 672–687. DOI: 10.1016/S0022-5371(82)90870-2

[19] Morrow, D. G., Greenspan, S. L., & Bower, G. H. (1987). Accessibility and situation models in narrative comprehension. Journal of Memory and Language, 26(2), 165–187. DOI: 10.1016/0749-596X(87)90122-7

[20] Oppenheim, G. M. (2013). Inner speech as a forward model? Behavioral and Brain Sciences, 36(4), 369–370. DOI: 10.1017/S0140525X12002798 PMC10913689 23789938

[21] Ostarek, M., & Huettig, F. (2017). A task-dependent causal role for low-level visual processes in spoken word comprehension. Journal of Experimental Psychology: Learning, Memory, and Cognition, 43(8), 1215–1224. DOI: 10.1037/xlm0000375 28114780

[22] Ostarek, M., & Huettig, F. (2019). Six challenges for embodiment research. Current Directions in Psychological Science, 28(6), 593–599. DOI: 10.1177/0963721419866441

[23] Pickering, M. J., & Garrod, S. (2013). An integrated theory of language production and comprehension. Behavioral and Brain Sciences, 36(4), 329–347. DOI: 10.1017/S0140525X12001495 23789620

[24] Rayner, K. (1998). Eye movements in reading and information processing: 20 years of research. Psychological Bulletin, 124(3), 372–422. DOI: 10.1037/0033-2909.124.3.372 9849112

[25] Scott, S. K. (2019). From speech and talkers to the social world: The neural processing of human spoken language. Science, 366(6461), 58–62. DOI: 10.1126/science.aax0288 31604302 PMC6858272

[26] Scott, S. K., Blank, C. C., Rosen, S., & Wise, R. J. (2000). Identification of a pathway for intelligible speech in the left temporal lobe. Brain: A Journal of Neurology, 123 Pt 12, 2400–2406. DOI: 10.1093/brain/123.12.2400 11099443 PMC5630088

[27] Stanfield, R. A., & Zwaan, R. A. (2001). The effect of implied orientation derived from verbal context on picture recognition. Psychological Science, 12(2), 153–156. DOI: 10.1111/1467-9280.00326 11340925

[28] Stites, M. C., Luke, S. G., & Christianson, K. (2013). The psychologist said quickly, “dialogue descriptions modulate reading speed!”. Memory & Cognition, 41(1), 137–151. DOI: 10.3758/s13421-012-0248-7 22927027 PMC3540141

[29] Tian, X., Zarate, J. M., & Poeppel, D. (2016). Mental imagery of speech implicates two mechanisms of perceptual reactivation. Cortex, 77, 1–12. DOI: 10.1016/j.cortex.2016.01.002 26889603 PMC5357080

[30] Vilhauer, R. P. (2016). Inner reading voices: An overlooked form of inner speech. Psychosis, 8(1), 37–47. DOI: 10.1080/17522439.2015.1028972

[31] Wildgruber, D., Ackermann, H., Kreifelts, B., & Ethofer, T. (2006). Cerebral processing of linguistic and emotional prosody: fMRI studies In Understanding Emotions, 156, 249–268. Elsevier DOI: 10.1016/S0079-6123(06)56013-3 17015084

[32] Yao, B. (2011). Mental simulations in comprehension of direct versus indirect speech quotations (Doctoral dissertation).

[33] Yao, B., Belin, P., & Scheepers, C. (2011). Silent reading of direct versus indirect speech activates voice-selective areas in the auditory cortex. Journal of Cognitive Neuroscience, 23(10), 3146–3152. DOI: 10.1162/jocn_a_00022 21452944

[34] Yao, B., Belin, P., & Scheepers, C. (2012). Brain “talks over” boring quotes: top-down activation of voice-selective areas while listening to monotonous direct speech quotations. Neuroimage, 60(3), 1832–1842. DOI: 10.1016/j.neuroimage.2012.01.111 22306805

[35] Yao, B., & Scheepers, C. (2011). Contextual modulation of reading rate for direct versus indirect speech quotations. Cognition, 121(3), 447–453. DOI: 10.1016/j.cognition.2011.08.007 21906731

[36] Yao, B., & Scheepers, C. (2015). Inner voice experiences during processing of direct and indirect speech In L. Frazier & E. Gibson (Eds.), Explicit and implicit prosody in sentence processing, 46, 287–307. Cham: Springer International Publishing DOI: 10.1007/978-3-319-12961-7_15

[37] Yao, B., & Scheepers, C. (2018). Direct speech quotations promote low relative-clause attachment in silent reading of English. Cognition, 176, 248–254. DOI: 10.1016/j.cognition.2018.03.017 29609099

[38] Zatorre, R. J., Belin, P., & Penhune, V. B. (2002). Structure and function of auditory cortex: music and speech. Trends in Cognitive Sciences, 6(1), 37–46. DOI: 10.1016/S1364-6613(00)01816-7 11849614

[39] Zwaan, R. A. (2004). The immersed experiencer: Toward an embodied theory of language comprehension In Psychology of Learning and Motivation, 44, 35–62. New York: Academic Press DOI: 10.1016/S0079-7421(03)44002-4

[40] Zwaan, R. A., & Pecher, D. (2012). Revisiting mental simulation in language comprehension: six replication attempts. Plos One, 7(12), e51382 DOI: 10.1371/journal.pone.0051382 23300547 PMC3530580

[41] Zwaan, R. A., Stanfield, R. A., & Yaxley, R. H. (2002). Language comprehenders mentally represent the shapes of objects. Psychological Science, 13(2), 168–171. DOI: 10.1111/1467-9280.00430 11934002

