# Supplementary material for: Mental Simulations of Phonological Representations Are Causally Linked to Silent Reading of Direct Versus Indirect Speech
Source: J Cogn. 2021 Jan 8;4(1):6. doi: 10.5334/joc.141 (PMC7792465; doi:10.5334/joc.141)
Supplement: Appendix A. — Reading Materials (24 critical items + 12 fillers). [file joc-4-1-141-s1.pdf]

Appendix A. Reading Materials (24 critical items + 12 fillers)

DS=Direct Speech; IS=Indirect Speech; NS=Non-Speech. The critical tongue twisters are highlighted in bold text.

[Critical Items]

1. **This shop sells short socks with spots**

Adele and Bethany are shopping at Screaming Mimi's for their Halloween party this Friday. As Adele browses through the range of costumes on offer, her eyes are caught by some spotty socks.
[DS] She exclaims excitedly, "**This shop sells short socks with spots!**"
[IS] She exclaims excitedly that **this shop sells short socks with spots**.
[NS] She discovers excitedly that **this shop sells short socks with spots**.
Bethany has a look at them and thinks that they are perfect for their clown costumes.

2. **Jack's knapsack strap snapped**

A group of nature lovers are exploring in the Peak District and are stopped by a stream. As Jack jumps over the stream, his equipment and supplies suddenly fall into the water.
[DS] Trevor shouts from behind, "Gosh, **Jack's knapsack strap snapped!**"
[IS] Trevor shouts from behind that **Jack's knapsack strap snapped**.
[NS] Trevor witnesses from behind that **Jack's knapsack strap snapped**.
Having heard the shouting, everyone gather together to help recover Jack's gear.

3. **Mr. Snipe's wife's knife a swipe**

At Lancaster Castle, the Snipe household is hosting a feast for the local governors. The kitchen is hectic when the butler barges in with a dirty knife in his hand.
[DS] In a hurry, he orders, "Quickly! Give **Mr. Snipe's wife's knife a swipe.**"
[IS] In a hurry, he orders to quickly give **Mr. Snipe's wife's knife a swipe**.
[NS] In a hurry, he attempts to quickly give **Mr. Snipe's wife's knife a swipe**.
A maid takes the knife and gives the butler a clean one.

4. **She sells seashells by the seashore**

Police officers Gilbert and George are looking for a woman named Judith for witness statements for a murder case. They arrive at Great Yarmouth, the east coast of Norfolk, and ask about Judith in a local pub.
[DS] The barman says, "I know her- **she sells seashells by the seashore.**"
[IS] The barman says that he knows her and that **she sells seashells by the seashore**.
[NS] The barman knows her and shows them that **she sells seashells by the seashore**.
Gilbert and George thank the barman and leave for the seashore.

5. **The sick shepherd's sixth sheep is sick**

The shepherd in the village has fallen ill and his sheep roam the hills freely. The sheep marked with number six is having a seizure when Jim and Alistair pass by.
[DS] Startled, Jim calls out, **"The sick shepherd's sixth sheep is sick!** We need to tell him.**"**
[IS] Startled, Jim calls out that **the sick shepherd's sixth sheep is sick** and that they need to tell him.
[NS] Startled, Jim realises that **the sick shepherd's sixth sheep is sick** and that they need to tell him.
They hurry to the shepherd's house and inform him of the bad news.

6. **Light a night-light on a light night like tonight**

Marcus is an activist for sustainability and energy saving. Tonight is full moon and his girlfriend is shocked when he turns off all the lights in the house.
[DS] Marcus explains, **“**Well, there is no need to **light a night-light on a light night like tonight.”**
[IS] Marcus explains that there is no need to **light a night-light on a light night like tonight.**
[NS] Marcus thinks that there is no need to **light a night-light on a light night like tonight.**
His girlfriend is upset because she is scared of the dark.

7. Three sweet Swiss witches switch sweets with each other

Samantha and friends are visiting Disneyland in Switzerland over Halloween. At The Witches’ Mountain, Samantha discovers three witches who are exchanging sweets in their cottage.
[DS] She Beckons her friends over, shouting, “Look! **Three sweet Swiss witches switch sweets with each other.”**
[IS] She Beckons her friends over, shouting that **three sweet Swiss witches switch sweets with each other.**
[NS] She Beckons her friends over to show them that **three sweet Swiss witches switch sweets with each other.**
They then enter the cottage to buy some sweets from the witches.

8. Fat Frankie fries fresh fish franticly

Fat Frankie at Codfather fish and chip shop is famous for her temperamental style of cooking. Today, some guests ask Jules the waiter the secret of making the fish so tender and succulent.
[DS] Jules gives them a wink and whispers, **“**Well, apparently **Fat Frankie fries fresh fish franticly.”**
[IS] Jules gives them a wink and whispers that apparently **Fat Frankie fries fresh fish franticly**.
[NS] Jules gives them a wink and mimes how **Fat Frankie fries fresh fish franticly.**
The guests like Jules’s sense of humour and burst out laughing.

9. The green glass globes that glow greatly

Schoolgirl Alexia is shopping for her Christmas party with her mother in a local store. Alexia’s eyes light up as she comes across some beautiful glass globes among other ornaments.
[DS] She pokes her mother and begs, “Mommy, I want **the green glass globes that glow greatly!”**
[IS] She pokes her mother and begs that she wants **the green glass globes that glow greatly.**
[NS] She pokes her mother because she wants **the green glass globes that glow greatly.**
Her mother is not a fan of the colour green and refuses to buy them.

10. Susie’s sister sews shirts for soldiers

In honour of their granddad who fought in WWII, Susie and her sister opened a tailoring shop in the 1980s to support soldiers. Today at a charity event,
[DS] the host introduces the duo with emotions, “For over 30 years, Susie runs the shop and **Susie’s sister sews shirts for soldiers.”**
[IS] the host introduces the duo with emotions that for over 30 years, Susie runs the shop and **Susie’s sister sews shirts for soldiers.**
[NS] the host introduces the duo and learns that for over 30 years, Susie runs the shop and **Susie’s sister sews shirts for soldiers.**
Their determination and perseverance have impressed the audience.

11. Ken Dodd’s dad’s dog is dead

Teacher Helen is concerned because her pupil Ken Dodd has missed school today. She asks Ken's best friend Eli to see if he knows anything about Ken's absence.

[DS] In a hushed voice, Eli tells her, **"Ken Dodd's dad's dog is dead."**

[IS] In a hushed voice, Eli told her that **Ken Dodd's dad's dog is dead.**

[NS] It turns out that **Ken Dodd's dad's dog is dead.**

Helen feels sorry for Ken and decides to send him a card.

12. Betty Botter buys a bit of butter for breakfast

Betty Botter always goes to the shop round the corner before breakfast. Her nosy neighbour is curious and asks the shop owner what she is up to.

[DS] The shop owner replies, **"**Ah yes – every morning **Betty Botter buys a bit of butter for breakfast."**

[IS] The shop owner replies that every morning **Betty Botter buys a bit of butter for breakfast.**

[NS] The shop owner remembers that every morning **Betty Botter buys a bit of butter for breakfast.**

Betty's neighbour is disappointed that the information is not very interesting.

13. Peter Piper picked a peck of pickled peppers

The children gather around in a circle to show their teacher what they have collected from the Morrison's farmyard. Everyone takes turns to tell the group what their partner has collected.

[DS] Penny speaks proudly, **"**My friend **Peter Piper picked a peck of pickled peppers."**

[IS] Penny speaks proudly that her friend **Peter Piper picked a peck of pickled peppers.**

[NS] Penny is very proud that her friend **Peter Piper picked a peck of pickled peppers.**

Penny herself picked a bunch of bananas and several cucumbers.

14. Pickford's packers packed a packet of pickled pears

The head of MI5 feels utterly humiliated that his agent tipped the Army to stop a Pickford removal van on the M6 on suspicion of carrying nuclear explosives but found nothing. At the mission report meeting,

[DS] he roars at the agent, "Christ! **Pickford's packers packed a packet of pickled pears**, not nuclear weapons!"

[IS] he roars at the agent that **Pickford's packers packed a packet of pickled pears** rather than nuclear weapons.

[NS] he is furious with the agent because **Pickford's packers packed a packet of pickled pears** rather than nuclear weapons.

The agent is fired on the spot and his memory is wiped out before he leaves the MI5.

15. The great Greek grape growers grow great Greek grapes

Aaron is giving a tour to wine enthusiasts at the Persephone & Hades Grape farm in Greece. One of his guests asks Aaron which nation grows the finest grapes.

[DS] Aaron replies with pride, "**The great Greek grape growers grow great Greek grapes.**"

[IS] Aaron replies with pride that **the great Greek grape growers grow great Greek grapes**.

[NS] Aaron believes that **the great Greek grape growers grow great Greek grapes**.

The Greek grapes are exported to many parts of Europe for wine making.

16. Wayne Williams went to Wales to watch walruses

The BBC wants to hire nature photographer Wayne Walliams for the Wonders series programme. The director for the show visits his house but Mr Walliams is nowhere to be found.

[DS] His neighbour tells the director, **"Wayne Williams went to Wales to watch walruses."**

[IS] His neighbour tells the director that **Wayne Williams went to wales to watch walruses.**

[NS] It turns out that **Wayne Williams went to Wales to watch walruses.**

The director has no choice but to wait until he comes back.

17. Watching window washers wash Washington's windows with warm washing water

Ruby is waiting for her boyfriend Ethan to go for lunch outside the skyscraper The Washington Building. When Ethan arrives, he apologises and asks what she was doing while waiting.

[DS] Ruby replies with a chuckle, “Nothing really, I was merely **watching window washers wash Washington’s windows with warm washing water.**”

[IS] Ruby replies with a chuckle that she was merely **watching window washers wash Washington’s windows with warm washing water**.

[NS] Ruby chuckles because she was merely **watching window washers wash Washington’s windows with warm water**.

They then head off to the Italian restaurant across the street for lunch.

18. Seth Smith at Sainsbury's sells thick slick socks

On a winter’s day, Arthur comes to the conclusion that he needs some new socks. As he leaves the house, he asks his wife where he can find good quality socks in town.

[DS] His wife yells from the kitchen, “Oh, **Seth Smith at Sainsbury’s sells thick slick socks**!”

[IS] His wife yells from the kitchen that **Seth Smith at Sainsbury’s sells thick slick socks.**

[NS] He then remembers that **Seth Smith at Sainsbury’s sells thick slick socks.**

He gets in his car, finds the fastest route to Sainsbury’s on his navigation system and drives off.

19. Chester cheetah chews a chunk of cheap cheddar cheese

Chester zoo’s cheetah is famous for having an appetite for almost everything. The Adams family come to see the cheetah just as the cheetah is eating cheddar cheese.

[DS] Amused, the son shouts, “Ha! **Chester cheetah chews a chunk of cheap cheddar cheese**!”

[IS] Amused, the son shouts that **Chester cheetah chews a chunk of cheap cheddar cheese**.

[NS] The son finds it amusing that **Chester cheetah chews a chunk of cheap cheddar cheese**.

Some experts believe that the cheetah’s enormous appetite is due to a rare genetic mutation.

20. A proper cup of coffee from a proper copper coffee pot

Giovanni De Luca owns an independent Italian coffee shop that proves very popular in Didsbury. Entrepreneur Alexander J. Jones wants to invest in Giovanni’s coffee and asks him his coffee-making secret.

[DS] Giovanni laughs and explains, “You can only make **a proper cup of coffee from a proper copper coffee pot**.”

[IS] Giovanni laughs and explains that one can only make **a proper cup of coffee from a proper copper coffee pot**.

[NS] Giovanni laughs and shows him that one can only make **a proper cup of coffee from a proper copper coffee pot**.

The entrepreneur realises that he needs more cash to invest in these copper coffee pots.

21. Picky people pick Peter Pan Peanut Butter

Thousands of independent peanut butter makers bring their very best products to this year’s Peanut Butter Festival in Redbank Valley. At a Disney-themed stall, the owner is very proud of his Peter Pan range.

[DS] He proclaims loudly, “Look! Even **picky people pick Peter Pan Peanut Butter**.”

[IS] He proclaims loudly that even **picky people pick Peter Pan Peanut Butter**.

[NS] He believes that even **picky people pick Peter Pan Peanut Butter**.
To his surprise, the visitors are actually more interested in his Snow White range.

22. A snack stacker snacks stacked snacks

Teenager Andy is working as a snack stacker at Bona Foodie over the summer. The owner is furious when he finds out that Andy has been secretly snacking on his stocks.
[DS] He drags Andy to his office and thunders, "My shop will go bust soon if **a snack stacker snacks stacked snacks!**"
[IS] He drags Andy to his office and thunders that his shop will go bust soon if **a snack stacker snacks stacked snacks**.
[NS] He drags Andy to his office to lecture him otherwise his shop will go bust soon if **a snack stacker snacks stacked snacks**.
Andy promises that he won't do it again and that his parents will pay for the compensation.

23. Write Wright right because it is not right to write Wright 'Rite'

Crime drama writer Mr Wright wants his son to be a writer like him when he grows up. As he inspects his son's homework, he notices that his son spells his name as "Rite" instead of "Wright".
[DS] Mr Wright explains to his son firmly, "You need to **write Wright right because it is not right to write Wright 'Rite'.**"
[IS] Mr Wright explains to his son firmly that he needs to **write Wright right because it is not right to write Wright 'Rite'**.
[NS] Mr Wright realises that his son needs to **write Wright right because it is not right to write Wright 'Rite'**.
Mr Wright thinks that his son's English teacher is not doing a very good job.

24. Frank's fisher fishes on Friday for Frank's Friday fresh fried fish-fest

At South Pier in Blackpool, Frank's fish and chip shop hosts a fish-fest every week and has attracted many visitors. Some visitors had a very good experience and come back to ask how they make the fish so fresh in the fest.
[DS] The waitress smiles and replies, "Apparently **Frank's fisher fishes on Friday for Frank's Friday fresh fried fish-fest**."
[IS] The waitress smiles and replies that apparently **Frank's fisher fishes on Friday for Frank's Friday fresh fried fish-fest**.
[NS] The waitress smiles because apparently **Frank's fisher fishes on Friday for Frank's Friday fresh fried fish-fest**.
The fish on other days of the week, however, is not as fresh as a result.

[Filler Items]

1. A Blackwell bookstore had recently opened in Edinburgh. One day, Alexia, a young mother living nearby, came in and asked for advice. She said to the assistant, “I am looking for birthday presents for my son.” The assistant replied, “That's wonderful! How old is your son?”

2. Julie and Mark had been classmates and had not seen each other for years. Today, they met in the local supermarket and Julie started a conversation about career paths. She said to Mark, “My life has been amazing! After merely three years, I’m now a solicitor.” Mark was very happy for her but did not want to reveal that he himself was unemployed.

3. One of Melanie’s students, Jason, came into her office and said that he hadn’t been able to reach her that morning. Melanie was confused because she had been in her office the whole time. She frowned and said that there must be something wrong with her telephone. She tried to call maintenance and realised that the cable was not plugged in.

4. Colleen was having a busy day working at the department store. She was serving a customer who wanted to know where he could buy some cosmetics for his wife. Impatiently she replied that cosmetics were actually on the second floor. The customer was in fact the owner of the store and he was not impressed with Collean's service.

5. Nicole was having some pain in her knees, so she booked an appointment with the local GP. The doctor asked what the trouble was and she said her knees were a little bit sore. The doctor let her stand up and found that her knees looked a bit swollen. It turned out that there was quite a bit of fluid in her knees that needed to be removed.

6. It was Christmas Eve and everyone was drinking and dancing at the local pub. Judith was already quite tipsy when she noticed that George was with a girl she had not seen before. She was very surprised because he had never told her that he had such a beautiful sister. It turned out that George's sister was adopted by his parents when she was 5 years old.

7. In order to finish the project on time, Audrey had been working in the office from 7am to 8pm without taking a break. Her colleague Sean had just come back from dinner with a big smile on his face, which upset her a bit. She complained, “It’s so unfair! My stomach has been rumbling all day. I could eat a horse.” Sean made a funny face and said, “Now you've mentioned it - I definitely tasted horse in my beef burger.”

8. Ramona and Keith were postgraduate students in linguistics. One afternoon, they met in the local café and Ramona started chatting about her favourite subjects. She said enthusiastically, “Latin is boring! I’m much more interested in Eastern languages.” Keith were not so much into Eastern languages, but he hated Latin, too.

9. Jasmine had been ill for a week so her brother Colin took her to the doctor. After an hour, Jasmine came back to the waiting room looking rather disgruntled. She complained to her brother that the treatment was absolutely useless. She added that next time, she would definitely go for a specialist.

10. At House of Fraser, the manager was demonstrating to her employees how to describe, in one sentence, the special features of a product. She did it by performing a mock sales pitch in front of them. Holding up a jacket, she said that it was made of the finest materials and represented the latest Italian couture. The employees were impressed with her delivery and were eager to give it a go themselves.

11. Adrian’s favourite shop was the 'Pound World' round the corner from him. This shop had everything, from fresh produce to birthday cards to screwdrivers. Everything was one pound. Usually, he got very good deals there but his best friend questioned whether he really needed all that junk. Apparently, Adrian's living room was filled with stuff from Pound World.

12. It was Sunday afternoon when Heather finally came back home after visiting her parents. She was outraged to find her boyfriend Ben sleeping like a pig in his messy bedroom where she couldn’t even find a space to stand. She could not believe how messy he was as she stepped on his dirty pyjamas lying on the floor. She dragged Ben out of bed and demanded him to tidy up or he would have no dinner tonight.
